# Supplementary material for: Integrating single-cell biophysical and transcriptomic features to resolve functional heterogeneity in mantle cell lymphoma
Source: Sci Adv. 2025 Dec 5;11(49):eady2963. doi: 10.1126/sciadv.ady2963 (PMC12680032; doi:10.1126/sciadv.ady2963)
Supplement: Supplementary file 1 — Figs. S1 to S16 Legends for tables S1 to S3 Tables S4 and S5 References [file sciadv.ady2963_sm.pdf]

Science Advances
AAAS

Supplementary Materials for

Integrating single-cell biophysical and transcriptomic features to resolve functional heterogeneity in mantle cell lymphoma

Ye Zhang *et al.*

Corresponding author: Scott R. Manalis, srm@mit.edu; Mark A. Murakami, mark_murakami@dfci.harvard.edu

Sci. Adv. **11**, eady2963 (2025)
DOI: 10.1126/sciadv.ady2963

The PDF file includes:

Figs. S1 to S16
Legends for tables S1 to S3
Tables S4 and S5
References

Other Supplementary Material for this manuscript includes the following:

Tables S1 to S3

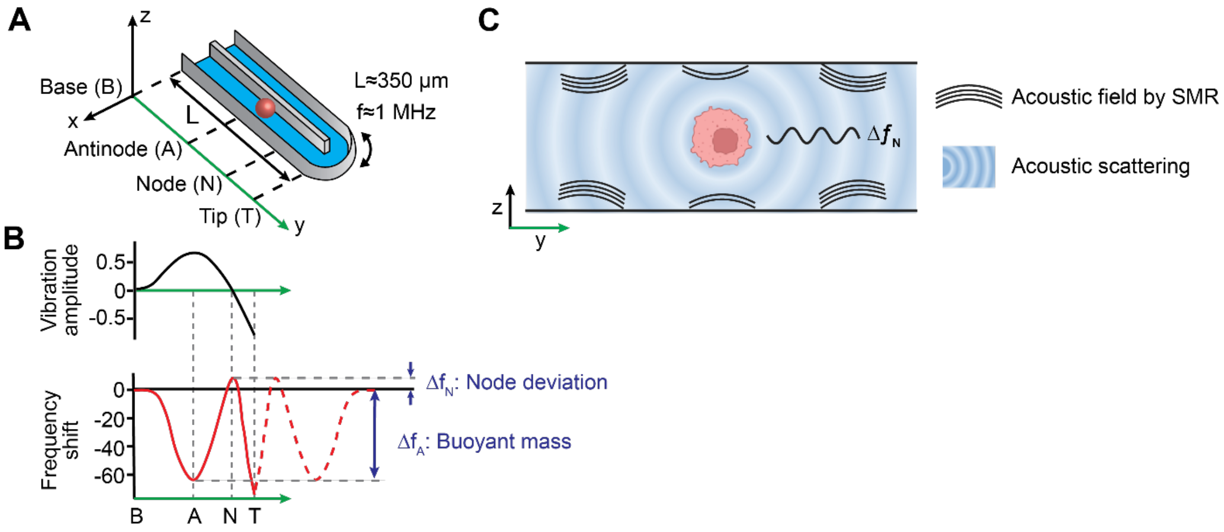

Fig. S1. Single-cell biophysical measurements utilizing the SMR. A) Schematic of the SMR with a cell flowing through the embedded fluid channel along the cantilever, which vibrates at resonant frequency f. **B)** Top, normalized vibration amplitude at the second mode. Bottom, resonant frequency shift when a single cell flows along the cantilever in the SMR. The vertical dashed lines indicate the positions of the cell along the cantilever, as in the schematic shown in (A). Buoyant mass is measured at the antinode (A), and node deviation is measured at the node (N). **C)** Illustration of frequency shift due to acoustic scattering. When a cell flowing in the fluid channel interacts with acoustic fields (black waves) generated by the cantilever vibration at resonant frequency, the particle-fluid interaction causes acoustic scatterings (blue waves), which shifts the measured resonant frequency. Created in BioRender. Zhang, M. (2025) https://BioRender.com/gq4p706.

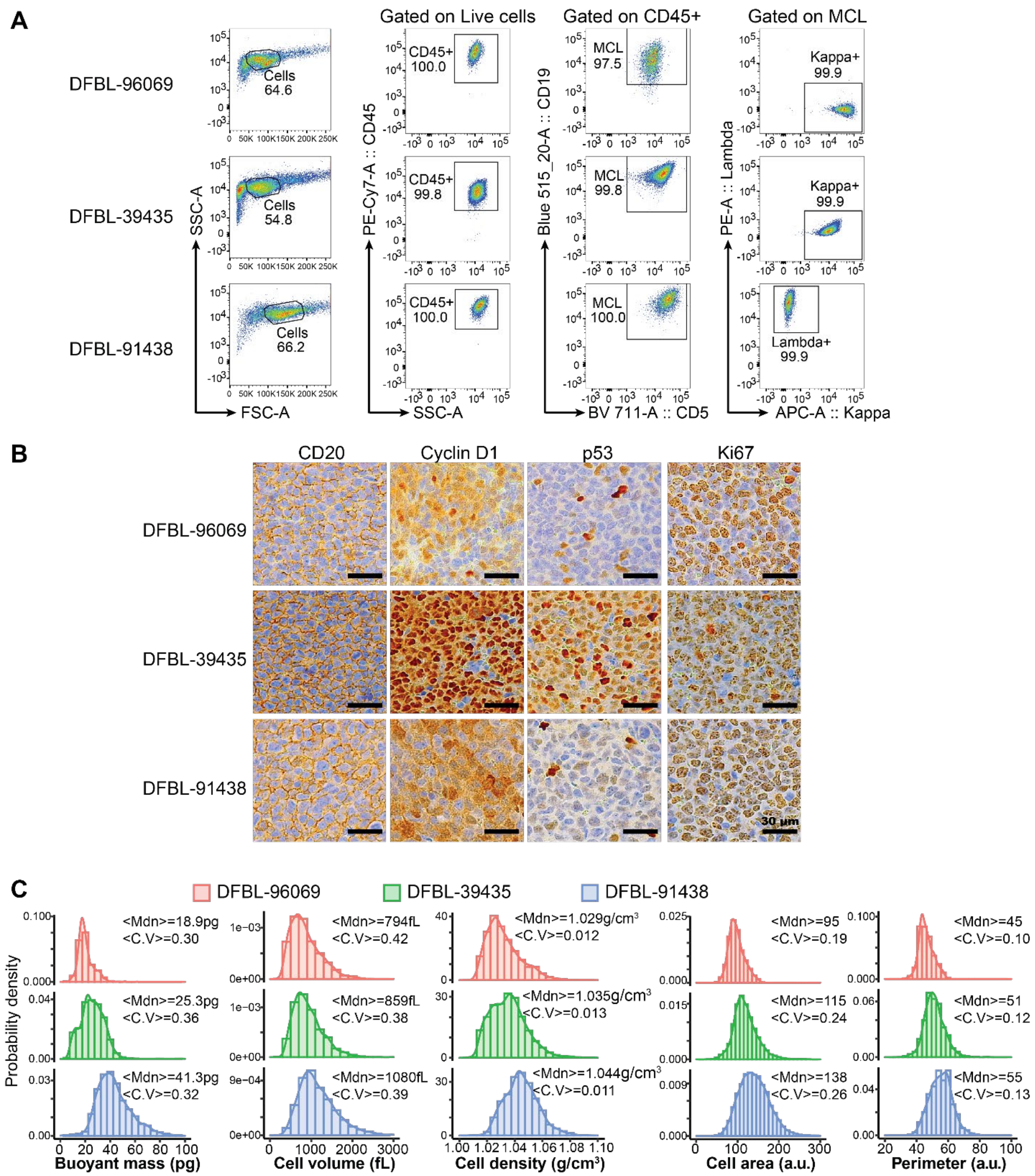

Fig. S2. Comprehensive immunophenotypic, histopathological, and biophysical characterization of three MCL PDX models. A) Flow cytometry analysis showing the immunophenotype of the three PDX models of MCL DFBL-96069, DFBL-39435, DFBL-91438. **B)** IHC images showing human CD20, cyclin D1, P53, and Ki67 expression in spleen cells from DFBL-96069, DFBL-39435, and DFBL-91438. All models express CD20, with strong cyclin D1 and Ki67 nuclear expression. DFBL-39435 shows strong p53 expression, while DFBL-96069 and DFBL-91438 have low TP53 levels. Images at 20X magnification, scale bar: 30 μm. **C)**

Measurement of single-cell buoyant mass, volume, and density of MCL cells from three PDX models was assessed using the fluorescence exclusion-coupled SMR. Total cell area and perimeter were evaluated with the Amnis® Imaging Flow Cytometer. The median (Mdn) and coefficient of variation (C.V) are indicated next to each graph.

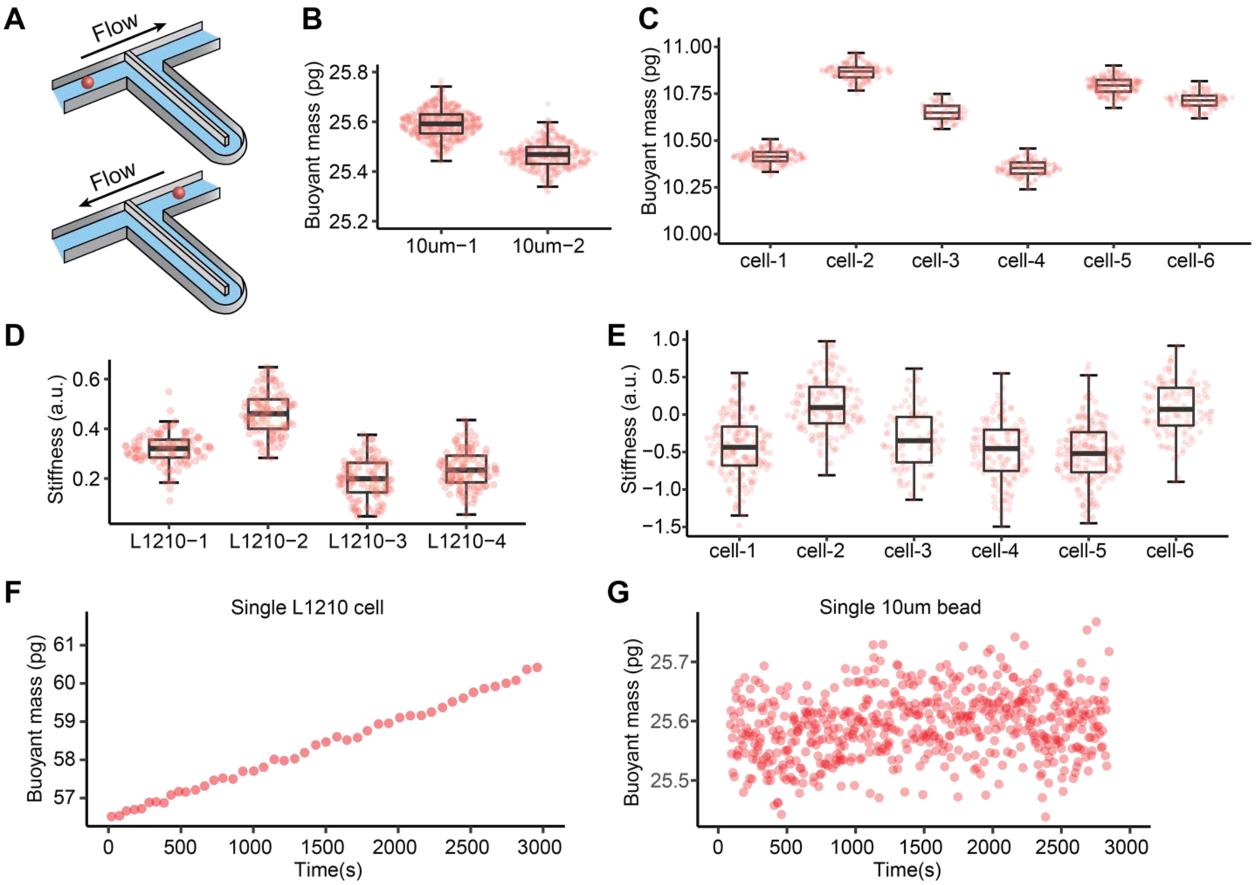

Fig. S3. Single-cell trapping on the SMR for repeated mass and stiffness measurement of an individual cell. **A)** Schematic of single bead/cell trapping on the SMR. Single-cell mass and stiffness is repeatedly measured as the bead/cell flows back and forth through the vibrating cantilever. To evaluate the technical interquartile ranges (IQRs) for mass measurements, we utilized 10 μm polystyrene beads **(B)** and naïve B cells isolated from human PBMCs **(C)**. To evaluate the IQRs for stiffness measurements, we used L1210 **(D)** and naïve B cells isolated from human PBMCs **(E)**. For assessing the technical IQR of mass measurements, the polystyrene bead is preferred over cell lines. This is because cell mass can vary during trapping **(F)** (*64*) —cells may accumulate mass as they grow or lose mass if apoptosis is induced —whereas the bead's mass remains constant throughout the experiment **(G)**. In contrast, when evaluating the technical IQR for stiffness measurements, cell-based samples provide a more representative range. Polystyrene beads are significantly stiffer than cells, meaning their stiffness values do not fall within the same range as those of the cells. The stiffness measurements from single-bead trapping data are available in our previous publication, Kang et al. (2019) (*24*). Additionally, since the technical IQR for stiffness differs between large and small cells, the L1210 cell line was chosen to represent larger cells (approximately 10–12 μm in diameter), while naive B cells represent smaller cells (approximately 6 μm in diameter).

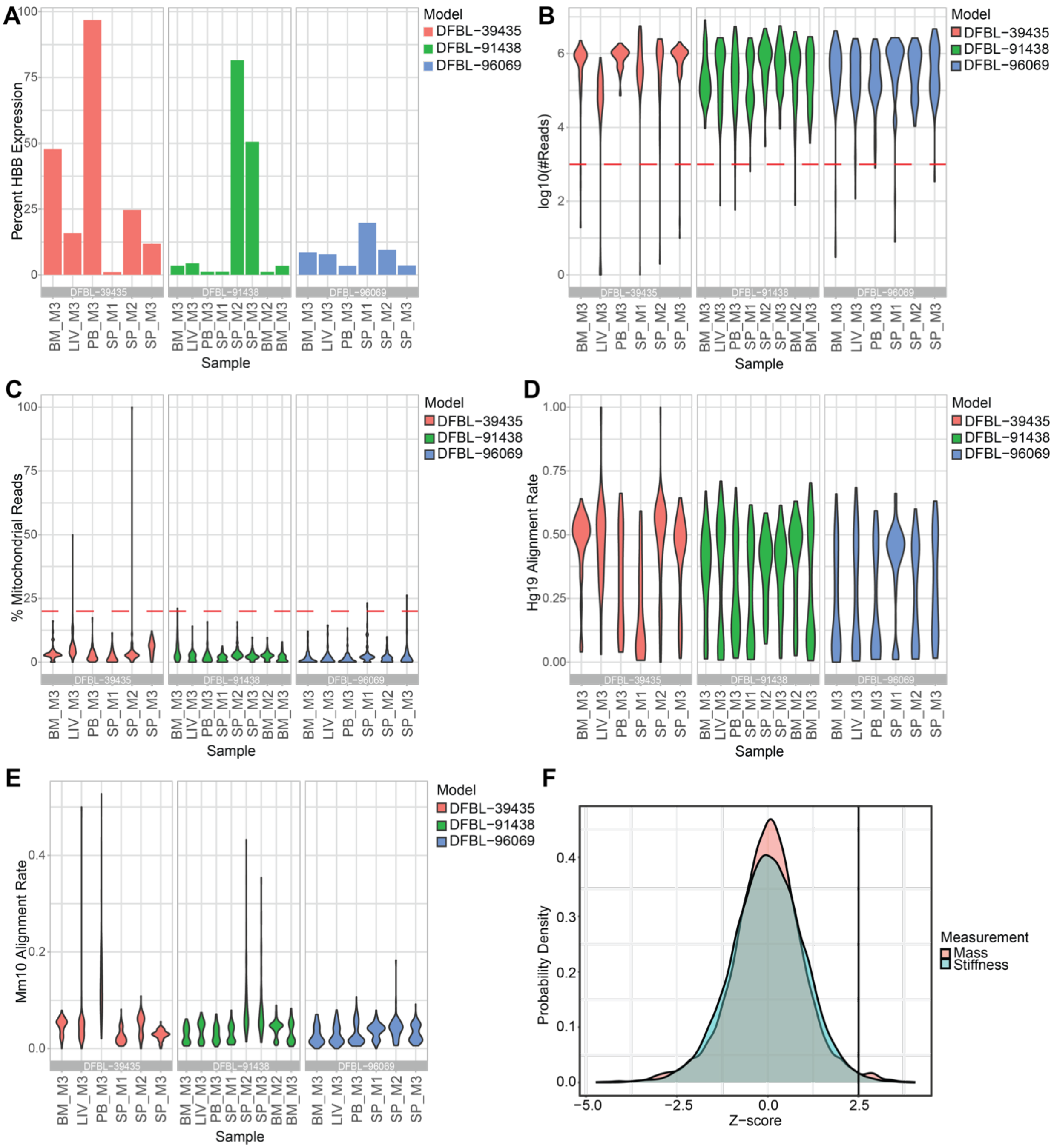

Fig. S4. Quality control metrics of scRNA-Seq data. A) Percentage of hemoglobin subunit beta (*HBB*) gene expression, **B)** Number of reads per cell, **C)** Percentage of mitochondrial reads, **D)** Alignment rate to human genome (Hg19), and **E)** Alignment rate to mouse genome (mm10) of human-enriched cells from DFBL-96069, DFBL-39435, DFBL-91438 isolated from different tissues (SP, spleen; PB, peripheral blood; BM, bone marrow; and LIV, liver). One to three biological replicates, each from a different mouse (M1, M2 or M3), were included per tissue. These metrics are used to assess sequencing depth, cell viability, and contamination, ensuring the integrity of the single-cell RNA sequencing data. **F)** Z-score distributions of genes correlated with cell mass and stiffness, and genes with z-score>2.5 are selected for ontology enrichment analysis.

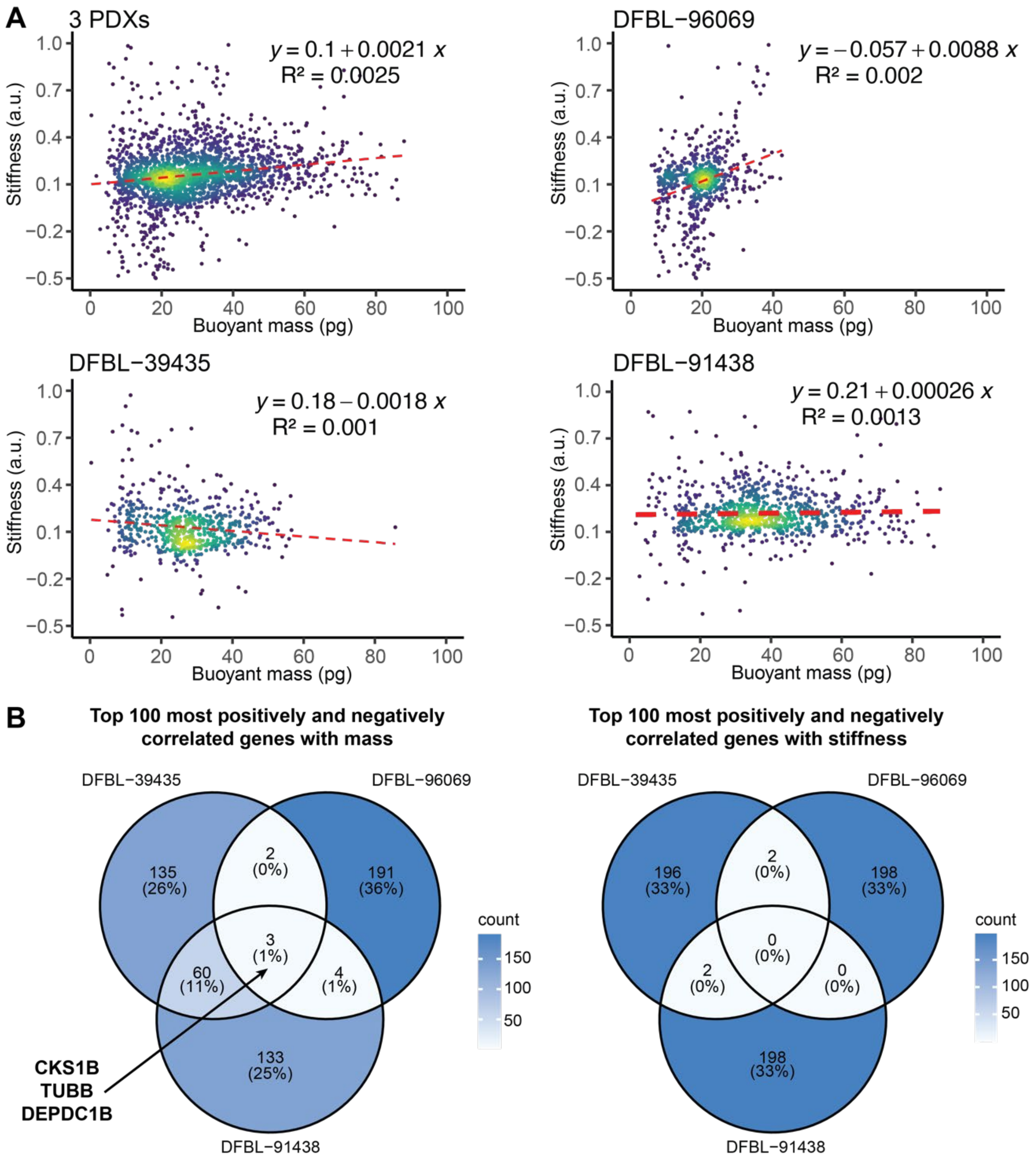

Fig. S5. Biophysical correlation between mass and stiffness within individual MCL PDX models and their associations with gene expression. A) Scatterplots show the relationship between buoyant mass and stiffness for individual MCL cells in three PDX models (DFBL-96069, DFBL-39435, and DFBL-91438), incorporating all cells measured from various tissues (spleen, peripheral blood, bone marrow, and liver) as shown in Figure 2. The linear regression fits (red dashed lines) and R^2 values indicate weak or negligible correlations within each model. Overlaid heatmaps highlight the density distributions of cells in the scatterplots. **B)** Venn diagrams show the number of shared genes across three PDX models via transcriptome-wide correlation analyses within each model, among the top 100 most positively and negatively correlated genes with mass (left) or stiffness (right).

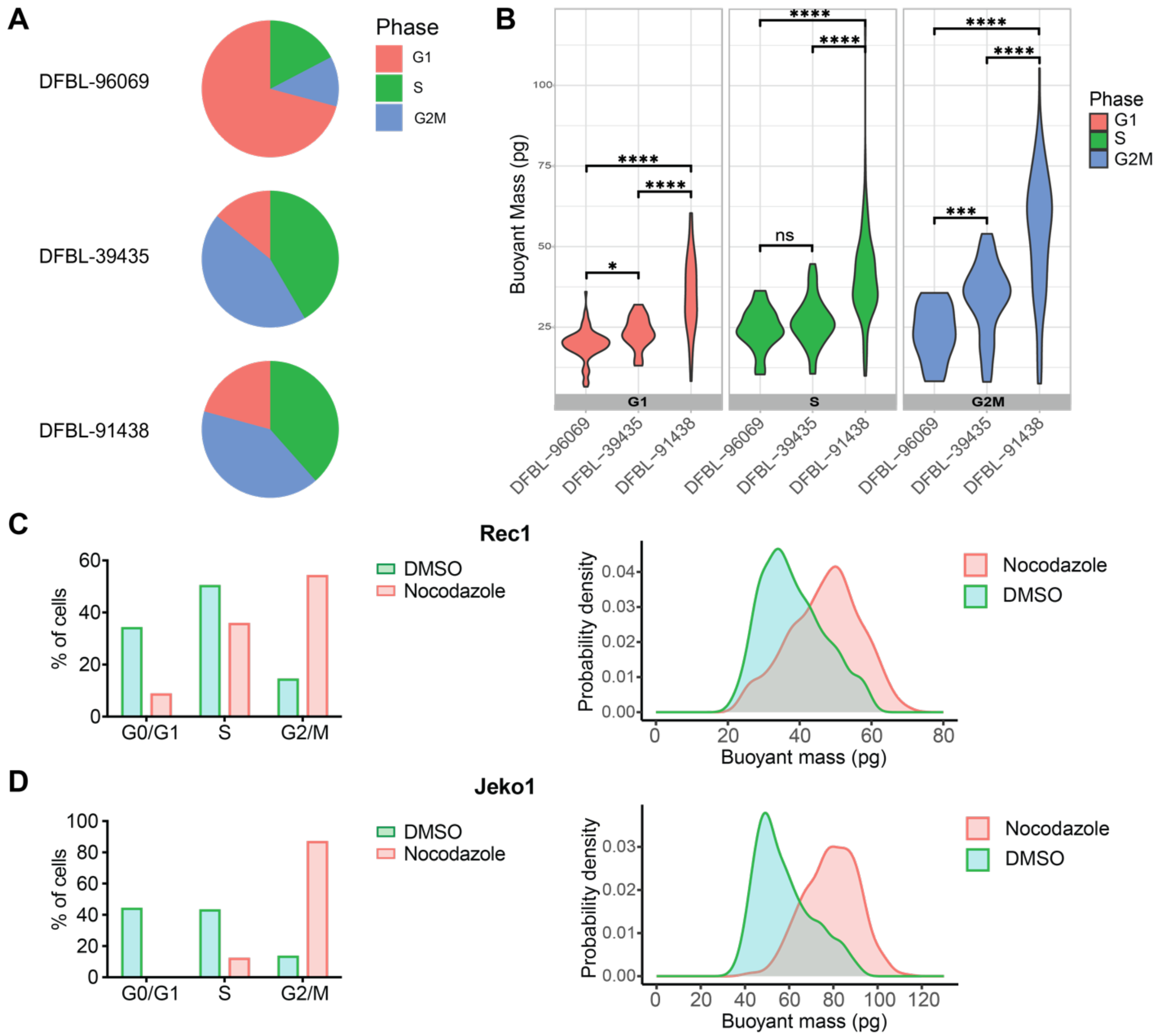

Fig. S6. Impact of cell cycle regulation on the buoyant mass of MCL cells. A) Pie charts summarizing the relative proportions of cells in each cell cycle phase for the three PDX models, illustrating variation in the cell cycle distribution across models. **B)** Violin plots displaying the distribution of buoyant mass for cells in different phases of the cell cycle (G0/G1, S, and G2/M) across three PDX models. C, D) Histograms showing changes in cell cycle distribution (left) by flow cytometry using PI staining and single-cell mass distribution (right) measured by the SMR after 20-hour treatment with 40 ng/mL nocodazole or DMSO in Rec1 **(C)** and Jeko1 **(D)** cells.

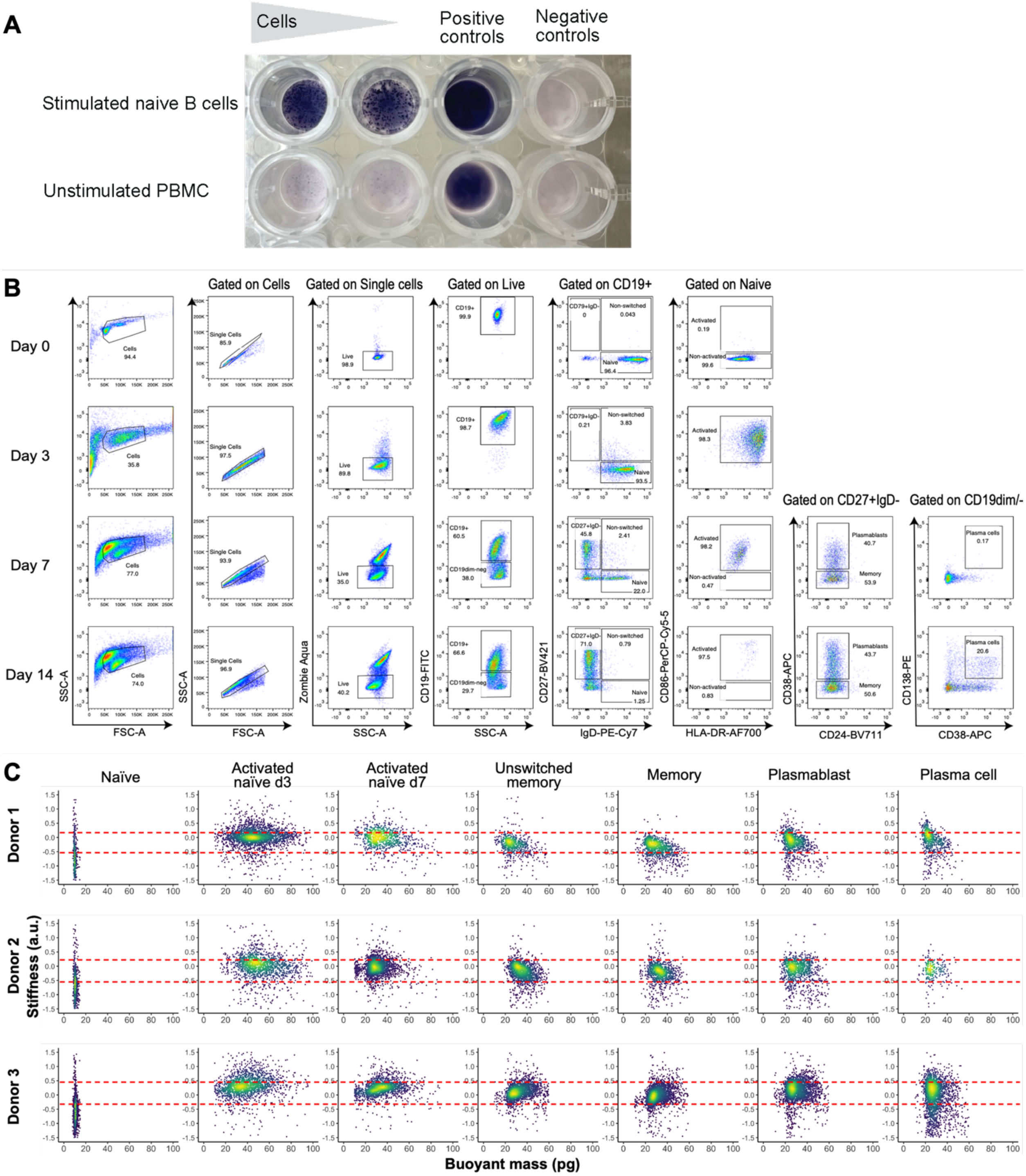

Fig. S7. Detection of IgG secretion and biophysical profiling of B cells across differentiation stages. A) Representative image of the ELISpot assay detecting total human IgG secretion in unstimulated PBMCs and stimulated naïve B cells cultured for seven days in RPMI + 10% FBS or the ImmunoCult™ human B cell expansion media respectively. Cells were incubated in the coated plate with RPMI + 10% FBS for 24 hours. Supernatant from the cell culture served as a positive control, while RPMI + 10% FBS medium was used as a negative control. **B)**

Representative gating strategy used to characterize human primary B-cells at various differentiation stages by flow cytometry following ex vivo activation. **C)** Buoyant mass vs stiffness profiles of single-cell B cells at different stages of differentiation isolated from three healthy donors.

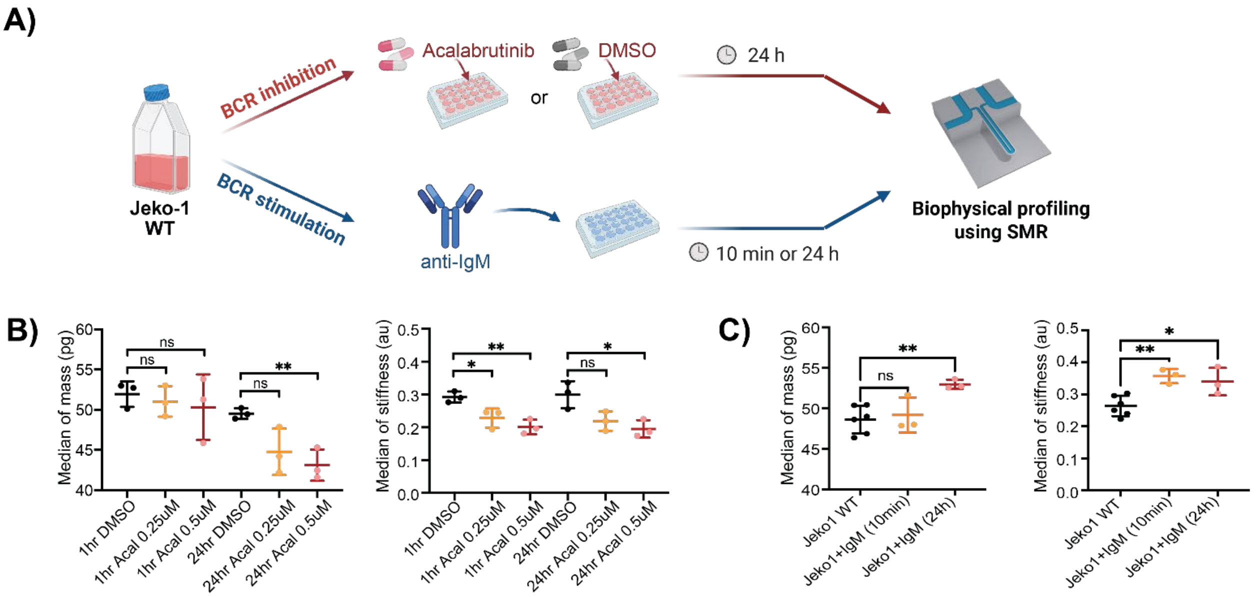

Fig. S8. BCR pathway perturbations drive changes in cell mass and stiffness in Jeko-1 cells. A) Schematic illustrating BCR inhibition via acalabrutinib treatment (top panel) and BCR stimulation through anti-IgM treatment (bottom panel) in Jeko-1 wild-type (WT) cells for biophysical profiling. Created in BioRender. Zhang, M. (2025) https://BioRender.com/2vjq1eu. **B)** Median single-cell mass and stiffness measurements obtained using the SMR from >500 Jeko-1 wild-type cells after 1h or 24h of ex vivo treatment with DMSO, 0.25 µM, or 0.5 µM acalabrutinib. Data are presented as the median ± SD of three biological replicates. **C)** Median single-cell mass and stiffness measurements from >500 Jeko-1 wild-type cells, with or without IgM stimulation for 10min or 24h, assessed using the SMR. Data represents the median ± SD of three biological replicates. NS: non-significant, $*P < 0.05$, $**P < 0.01$ as compared between indicated groups (Student's t-test).

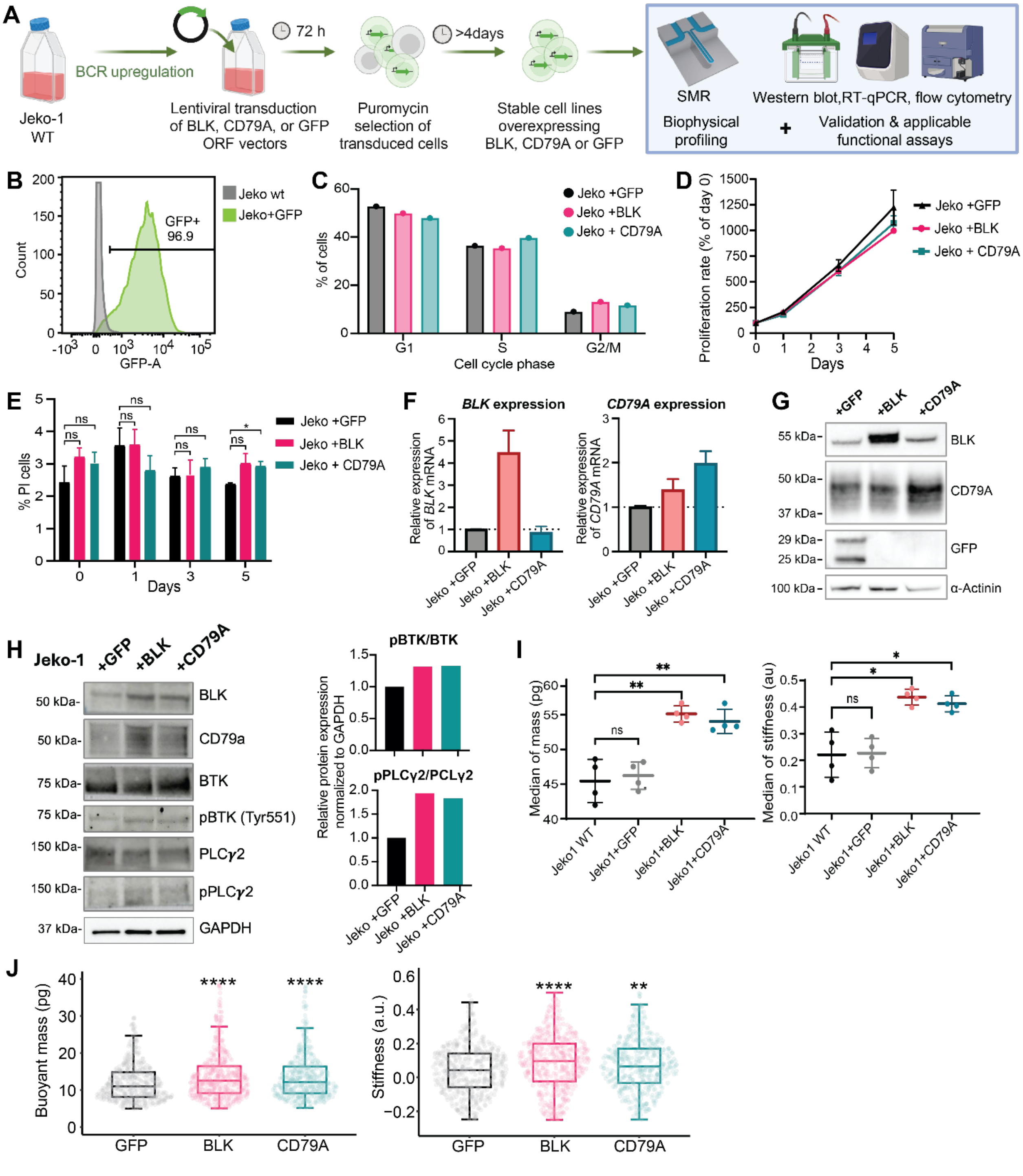

Fig. S9. Functional analysis of BLK and CD79A overexpression in Jeko-1 cells. A) Schematic illustrating BCR pathway enhancement through BLK and CD79A overexpression in Jeko-1 wild-type (WT) cells for biophysical profiling. Stable overexpression in cell lines was validated using RT-PCR, western blot, and flow cytometry. Functional assays (cell cycle and proliferation) further assessed GFP, BLK or CD79A-expressing cells. Created in BioRender. Zhang, M. (2025) https://BioRender.com/q6wsjha. **B)** FACS analyses of GFP staining in Jeko1^{+GFP} cells in comparison to Jeko-1 wild type cells. **C)** Representative cell cycle distribution measured by flow

cytometry following propidium iodine (PI) staining. **D)** Cell proliferation of Jeko-1 cells overexpressing GPF, BLK or CD79A over 5 days, as measured by flow cytometry. Data are presented as mean ± SEM of n= 3. **E)** Histograms showing the mean ± SEM of the percentage of PI positive cells measured by flow cytometry (n=3). **F)** Relative mRNA expression of *BLK* and *CD79A* measured by RT-qPCR in Jeko-1 overexpressing GFP, BLK, or CD79A. Results are presented as fold change normalized to *GAPDH* mRNA. Each bar represents the mean ± SEM of two independent stable cell lines. **G)** Representative images of western blot showing BLK, CD79A, GFP and α-actinin protein in Jeko-1 overexpressing GFP, BLK or CD79A. **H)** Representative images of western blot showing downstream effectors of CD79A and BLK proteins in Jeko-1 overexpressing GFP, BLK or CD79A following LPS treatment. One representative of two western blots is shown. Quantification of the relative expression levels of phospho-BTK (pBTK/BTK) and phospho-PLCγ2 (pPLCγ2/PLCγ2) are shown on the right. **H)** Median single-cell mass and stiffness measurements obtained using the SMR from >500 cells of Jeko-1 wild-type and Jeko-1 cells stably overexpressing GFP, BLK or CD79A. Data are presented as median ± SD of three biological replicates. **J)** Single-cell mass and stiffness measurements obtained using the SMR from >500 Jeko-1 cells overexpressing GFP, BLK or CD79A following LPS treatment. One representative experiment from two biological replicates is shown. ****P < 0.0001, **P < 0.01 as compared between indicated groups (Student's t-test).

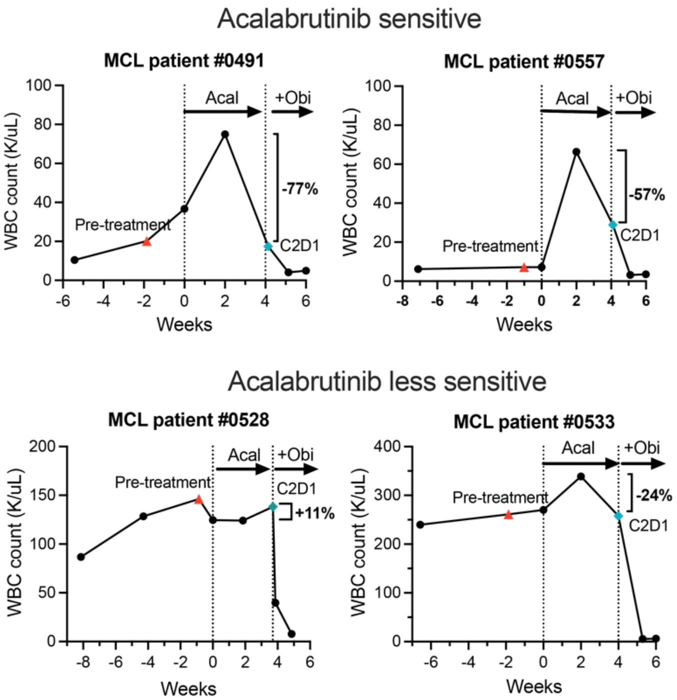

Fig. S10. White blood cell dynamics of acalabrutinib-treated patients with MCL. Total white blood cell (WBC) count over time in the acalabrutinib sensitive and less sensitive patient groups. The patients were treated for 4 weeks with acalabrutinib (Acal) alone before the addition of obinutuzumab (Obi). All patients except #0528 experience lymphocytosis after the start of acalabrutinib. The percent WBC reduction on Acal is indicated and was calculated from the peak level within the 30 days of Acal treatment to the end of Acal monotherapy. The pre-treatment samples (red triangle) and the Cycle 2 day 1 (C2D1; blue diamond) samples were used in our study.

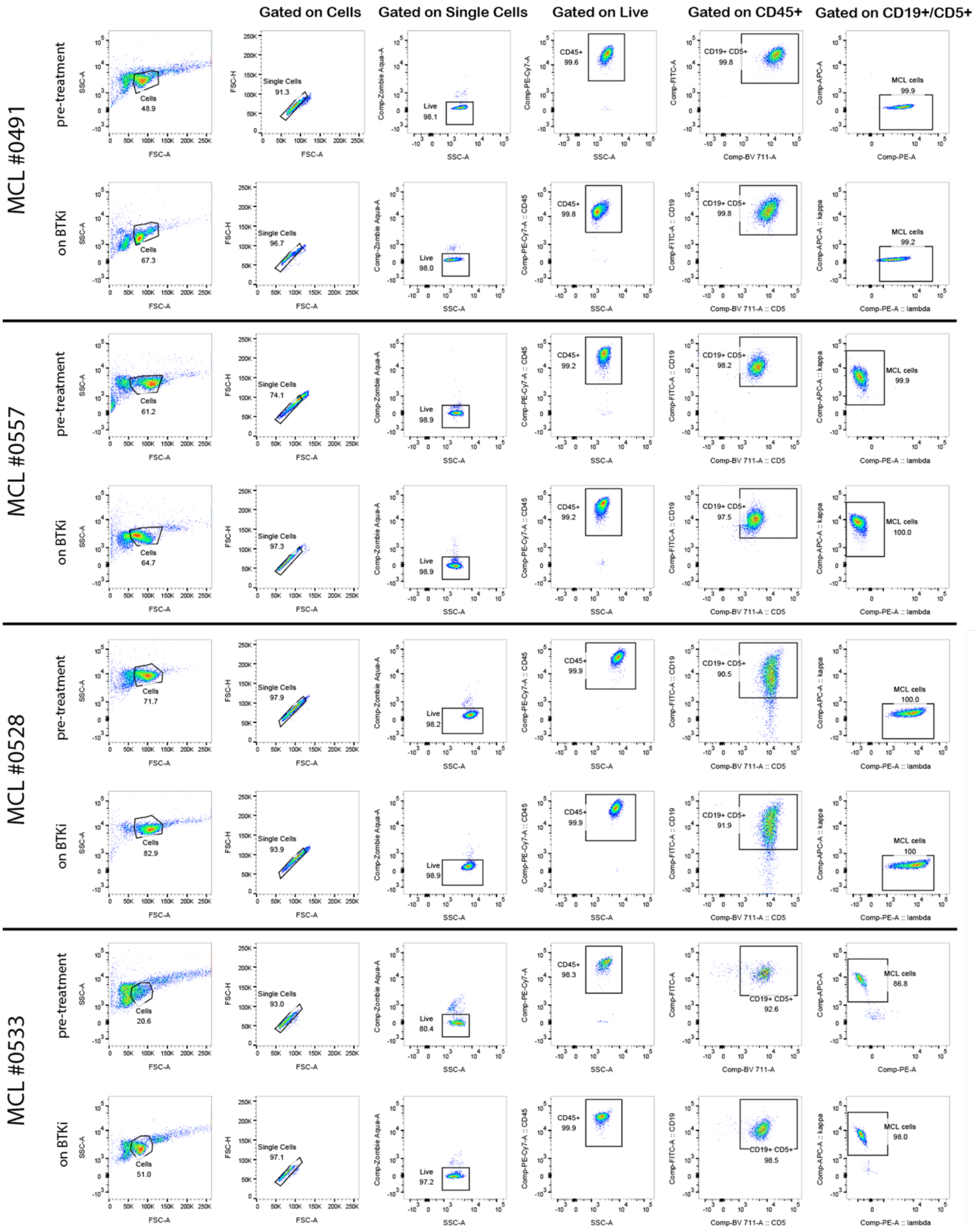

Fig. S11. Flow cytometry of the MCL primary samples. Flow cytometry panels used to characterize MCL tumor cells following human B cell enrichment. Cells were isolated from patients with MCL at pre-treatment or after four weeks of treatment with the BTKi acalabrutinib.

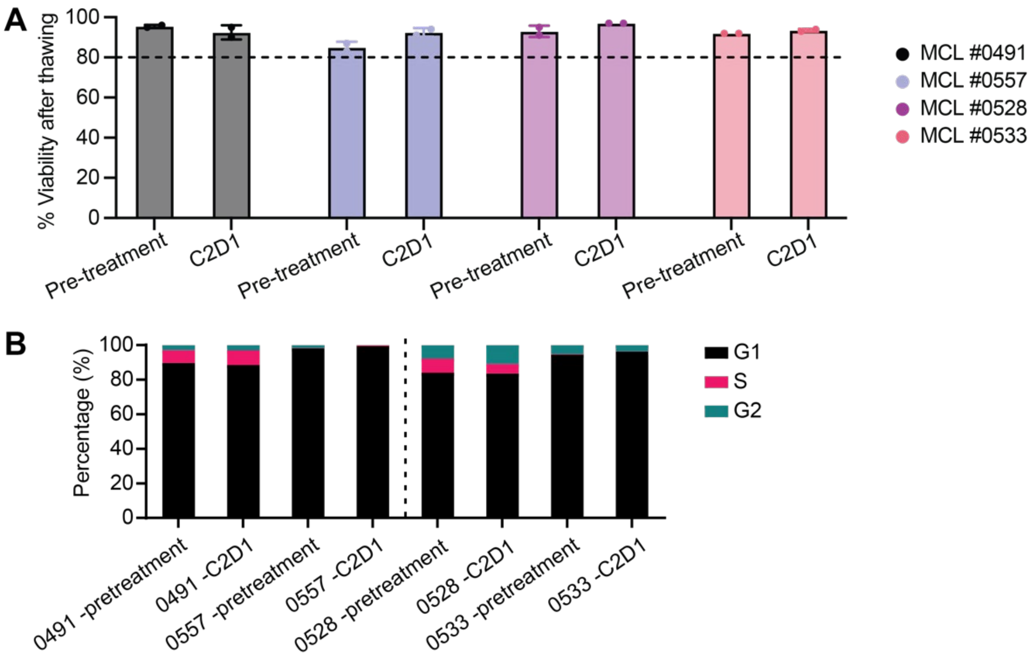

Fig. S12. Cellular viability and cell cycle distribution in acalabrutinib-treated patients with MCL. A) Percentage of viable cells measured by trypan blue after thawing. **B)** Cell cycle distribution of the cells analyzed by flow cytometry following propidium iodide staining after thawing.

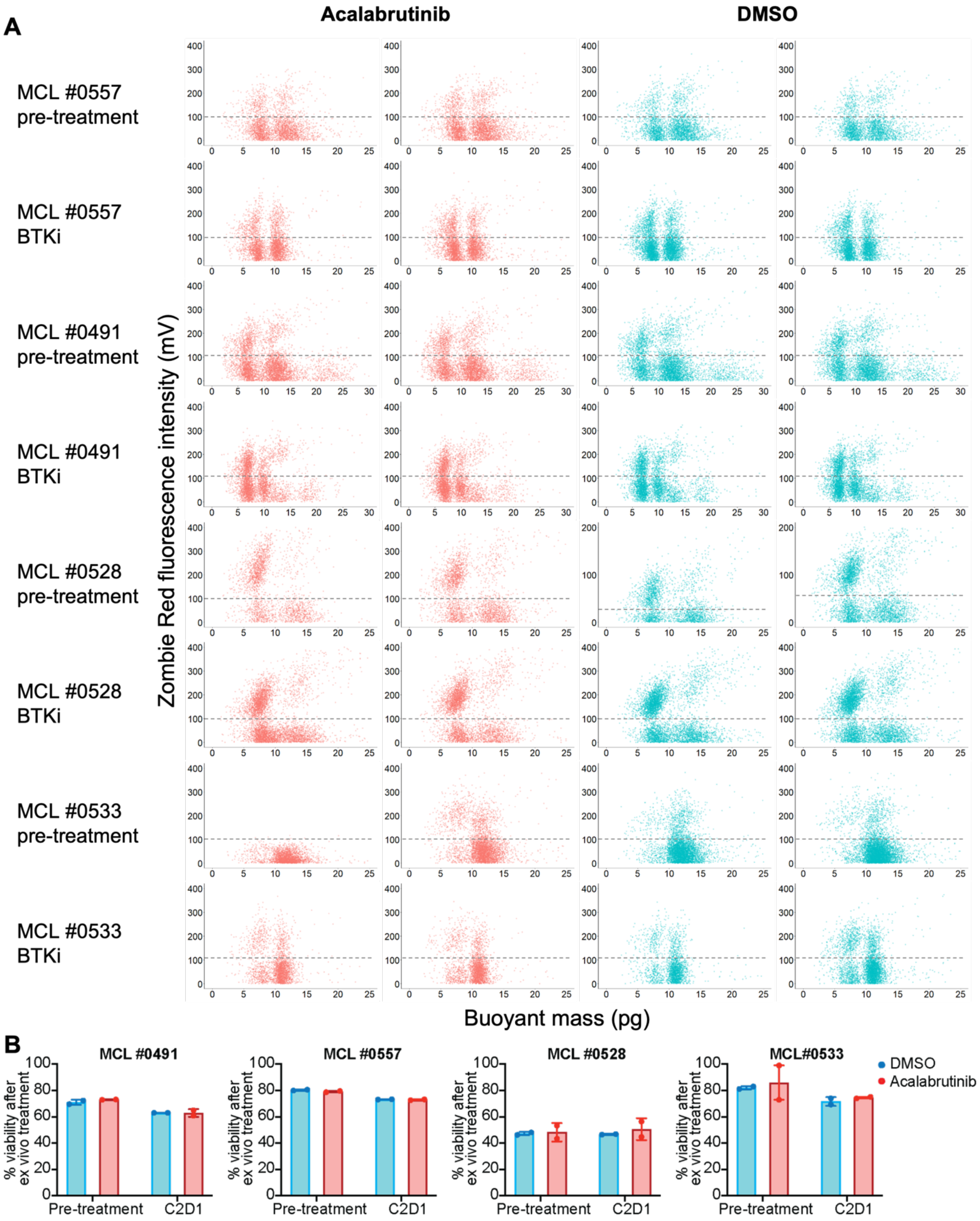

Fig. S13. Paired mass-viability assessment of MCL cells after ex vivo drug treatment via the fluorescence exclusion-coupled SMR. A) Paired single-cell measurement of buoyant mass (x-axis) and cell viability (indicated by ruby fluorescence intensity on the y-axis) following zombie

red staining for MCL cells from AVO patient samples. Analyses were conducted on samples collected at pre-treatment and four weeks of *in vivo* treatment with the BTKi acalabrutinib. Cells were subsequently treated *ex vivo* in duplicate with either acalabrutinib or DMSO for 24 hours and analyzed using the fluorescence-coupled SMR. **B)** Percentage of viable MCL cells following *ex vivo* drug treatment, as measured by negative zombie red staining from panel A.

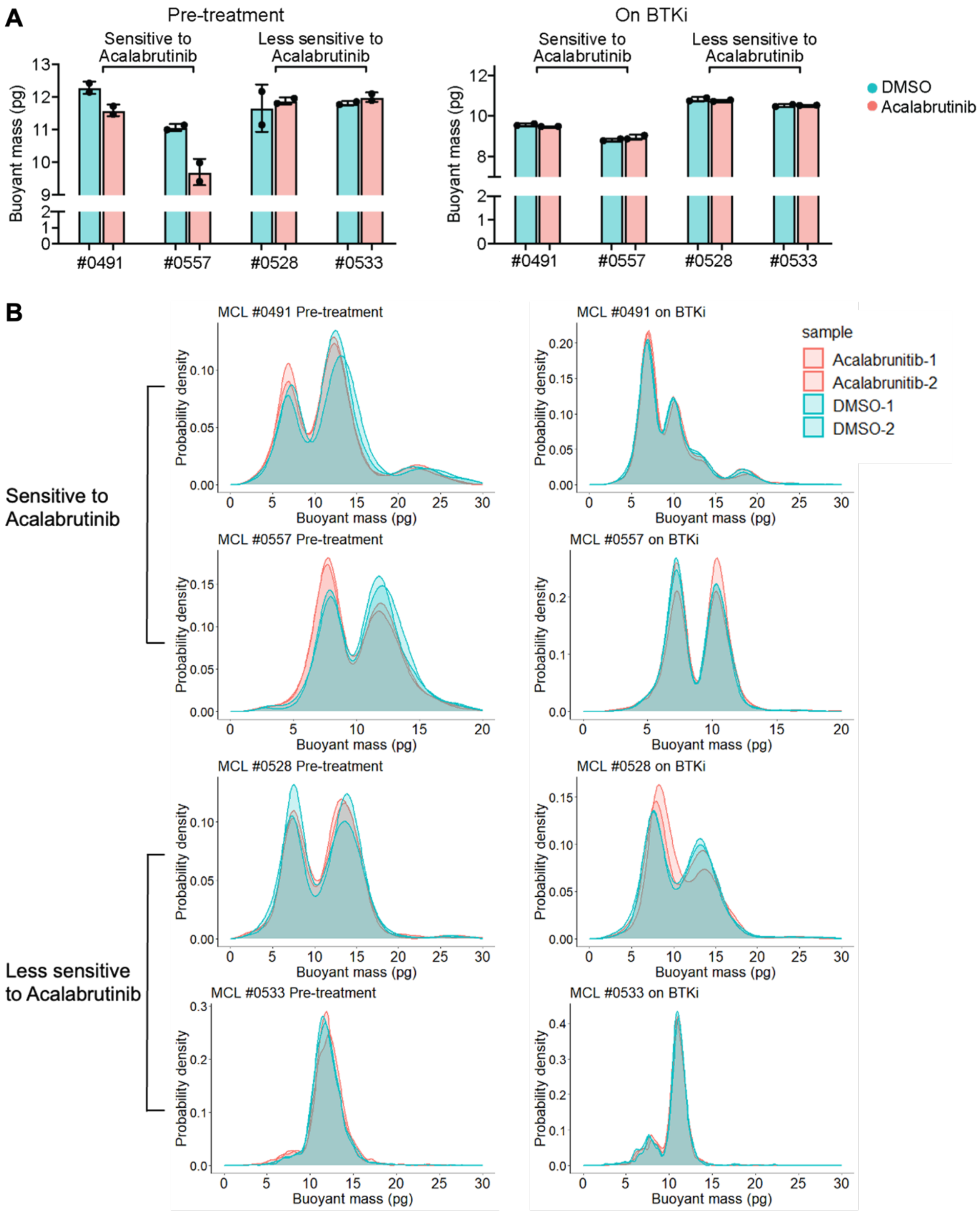

Fig. S14. Buoyant mass profiles of MCL patient samples after ex vivo acalabrutinib treatment. Histograms showing **A)** the median mass or **B)** the single-cell mass distribution of MCL primary samples of two acalabrutinib-sensitive and two less-sensitive MCL primary samples at pre-treatment (left) and after four weeks of *in vivo* treatment with acalabrutinib (on BTKi) (right). Cells were subsequently treated *ex vivo* in duplicate with either acalabrutinib or DMSO for 24 hours and analyzed using the fluorescence-coupled SMR.

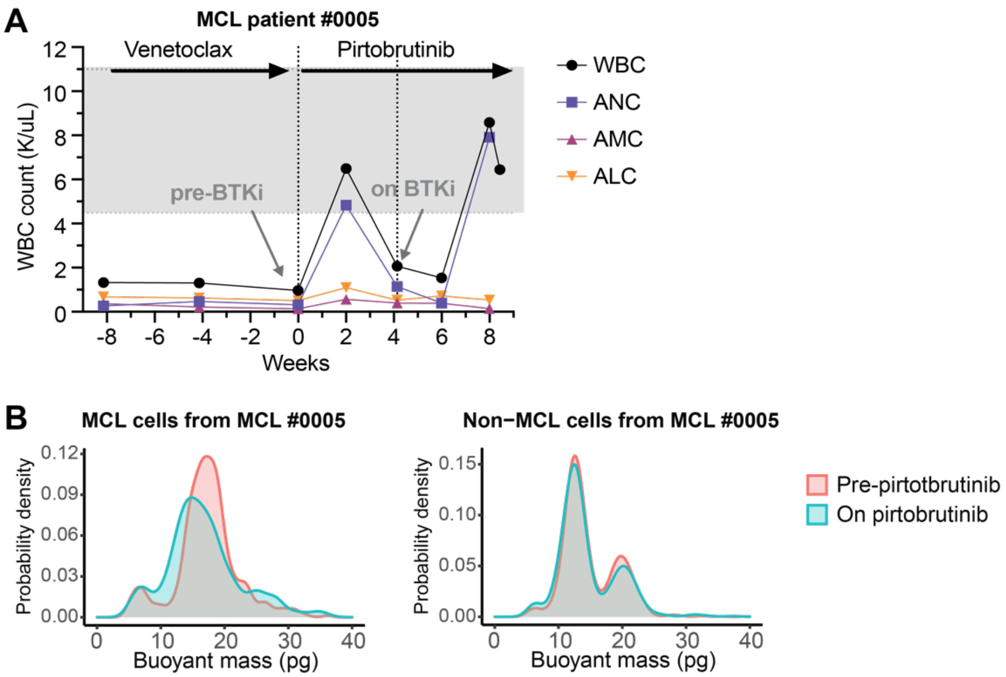

Fig. S15. Peripheral blood dynamics and single-cell mass shifts in response to pirtobrutinib in MCL. A) Total WBC, absolute neutrophil count (ANC), absolute monocyte count (AMC) and absolute lymphocyte count (ALC) over time in a MCL patient pre- and post- pirtobrutinib treatment. At the pre-pirtobrutinib timepoint, strong evidence of active disease progression led to a change in therapy. After one cycle of pirtobrutinib, the patient exhibited signs of a transient clinical response, including recovery of neutrophils, as well as symptomatic improvements with weight gain, increased energy, and enhanced appetite. **B)** Single-mass measurements of serial peripheral blood specimens from a patient with relapsed/refractory MCL collected at pre- and post-BTKi following four weeks of pirtobrutinib treatment. MCL and non-MCL cells were enriched using FACS.

A

SAMPLE ID	SAMPLE TYPE	AGE AT SAMPLE	SEX	CYTOGENETICS	IGHV	*TP53*	PREVIOUS TREATMENTS BEFORE ACALABRUTINIB
**JB-0215 PRE-TREATMENT**	PBMC	66	M	90.5% 13q	unmutated	Wild type	BR+ Lenalinomide
**JB-0215 PROGRESSION**	PBMC	70	M		unmutated	Mutated	
**JB-0375 PRE-TREATMENT**	PBMC	50	M	34.5% 11q deletion, 20.5% 13q deletion, 19% 17p deletion	unmutated	Wild type	FCR
**JB-0375 PROGRESSION**	PBMC	54	M			Wild type	
**JB-0097 PRE-TREATMENT**	PBMC	52	M	94% 11q del, 86% 13q del	unmutated	Wild type	FR, Rituxan, Spebrutinib, Ibrutinib
**JB-0097 PROGRESSION**	PBMC	57	M		unmutated	Wild type	

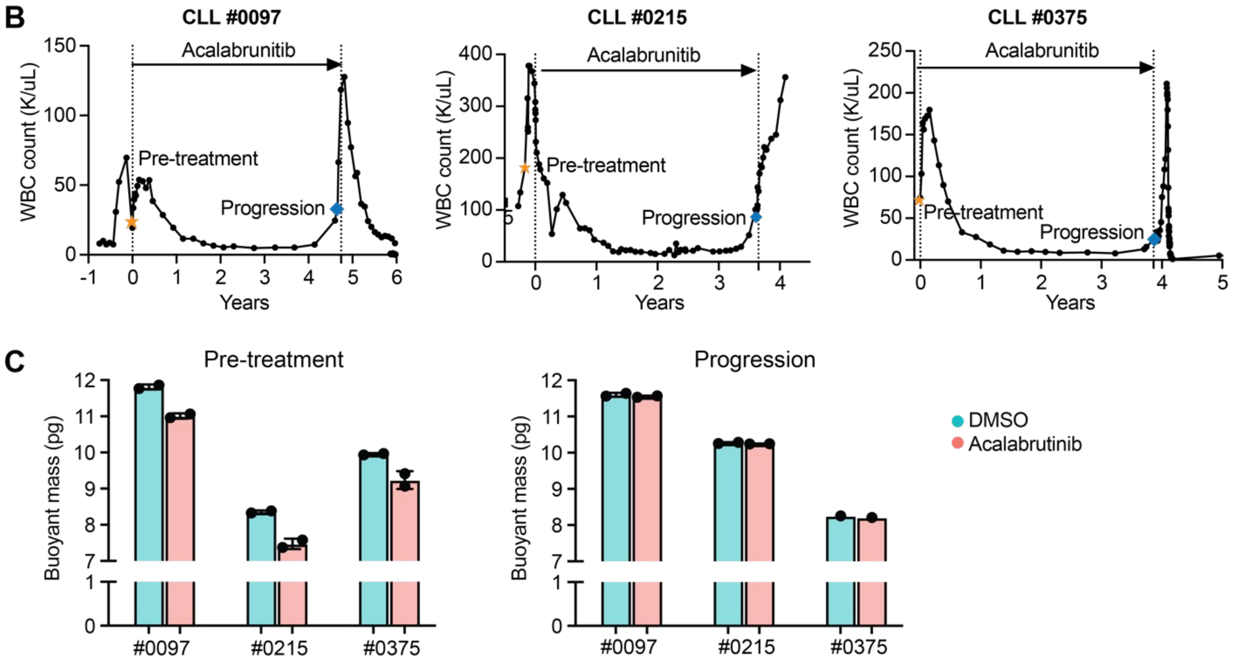

Fig. S16. Clinical information and biophysical analysis of CLL patient samples treated with acalabrutinib. A) Clinical and molecular characteristics of CLL patient samples. Bendamustine (B), rituximab (R), fludarabine (F), cyclophosphamide (C). **B)** WBC counts over time in CLL patients treated with acalabrutinib monotherapy. The pre-treatment (yellow triangle) and progression (blue diamond) samples were used in our study. **C)** Bar graphs showing the median mass measured by the SMR of three CLL primary samples at pre-treatment and progression timepoints, followed by 24 hours of ex vivo drug treatment in duplicate with DMSO or acalabrutinib.

Table S1. Mutational landscape of three MCL PDX models identified by hybrid capture, target enrichment next generation sequencing of coding exons of 205 genes. (separate file)

Table S2. Gene ontology results from GSEA analysis of top genes (z-score > 2.5) correlated with mass and stiffness across DFBL-39435, DFBL-96069, and DFBL-91438. (separate file)

Table S3. List of genes correlating with cell mass, cell stiffness and both mass and stiffness across DFBL-39435, DFBL-96069 and DFBL-91438. (separate file)

Table S4. MCL patient's clinical information.

Sample ID	#0491	#0557	#0528	#0533
**Sex-Age**	M-69	M-68	M-69	M-70
**Prior treatment for MCL**	Induction BR/R-AraC, maintenance R	None	None	None
**Prior ASCT**	Yes	No	No	No
**MCL stage**	III	IV	IV	III
**ECOG Performance Status**	1	1	1	0
**Histological subtype**	Classic	Classic	Classic	Classic
**Bone marrow involvement**	51%	7%	50%	76%
**Ki-67 index**	15-20%	<30%	40%	Not available
**TP53 status**	Mutated	Wild-type	Mutated	Mutated
**Cytogenetics**	46,XY,del(1)(p22p13), add(4)(q21), -10,t(11;14)(q13; q32), add(13)(q22),del(14)(q24q32)(q24q32),+mar[8] /46,idem,t(5;15)(q13;q22), add(10)(p13)[6]/46,XY[6] .nuc ish(CCND1,IGH)x3(CCND1 con IGHx2-3)[134/200]	46,XY,t(11;14)(q13;q32)[4]/ 46,idem,del(9)(q12q3?4)[cp5]/46, XY[11]	43,-Y,add(X)(p11.2),-9 ,-10,-11,t(11;14)(q13;q32),add(12)(p1?2),-13,-18,+2-4mar [cp4]/45,X,Y[4]/46, XY[12]	45,XY,t(11;14)(q13;q32), der(15;17)(q10;q10)[4]/45, idem,t(2;13)(p13;q12), -12, +mar[cp11]/46, XY[cp5]
**Pathogenic known mutations**	**PPM1D:** c.1528_1529insA (p.N512Kfs*16)7.1% VAF, c.1445delT(p.L482Rfs*3) 1% VAF; **TP53**: c.638G>A (p.R213Q) 66.2% VAF;	**NOTCH1**: c.7541_7542delCT (p.P2514Rfs*4) 5.3% VAF	**TP53:** c.524G>A (p.Rl75H) 95% VAF **ATM:** c.901+1G>C (splice site) 26.4% VAF	**CCND1**: c.130T>G (p.Y44D) 38.5% VAF; **TP53**: c.743G>A (p.R248Q) 65.7% VAF

	**ZRSR2**: c.1070_ 1106delTTGGGAAGA A CTCCGAAAGGAGG GAGAGGATGGGCC AinsGC(p.F357Cfs*16) 7.3% VAF			
**Copy number variations**	gain **SF3B1**, **IDH1** (on 2q); CN-LOH **TP53** (on 17p)	None detected	gain **RAD21**, **MYC** (on 8q); 1 copy deletion **JAK2**, **CDKN2A**, **CDKN2B** (on 9p); 1 copy deletion **ABL1**, **NOTCH1** (on 9q); gain **WT1** (on 11p); gain **ATM**, amplification **KMT2A**, 1 copy deletion **CBL** (on 11q); 1 copy deletion **ETV6**, **ETNK1**, **KRAS** (on 12p); 1 copy deletion **FLT3** (on 13q); gain **MAP2K1**, **IDH2** (on 15q); CN LOH **TP53** (on 17p)	1 copy deletion **DNMT3A** (on 2p), **PRPF8** (on 17p), **TP53** (on 17p)
**Variants of unknown significance**	**MPL**: c.655C>G (p.Q219E) 58.7% VAF	**CDKN2A**: c.106G>A (p.A36T) 41.7% VAF	**ATM:** c.8293G>A (p.G2765S) 41.2% VAF; **CREBBP:** c.1369A>G (p.I457V) 48% VAF	**TET2**: c.4787A>G (p.N1596S) 48.3% VAF

Table S5. Antibodies used for flow cytometry.

Antibody	Clone	Catalog #	Assay(s)
Pe/Cy7 anti-human CD45RA	HI100	BioLegend, #304125	PDX and primary cell characterization
BV711 anti-human CD5	L17F12	BD Biosciences, #742552	PDX and primary cell characterization
APC anti-human Kappa	TB28-2	BD Biosciences, #341098	PDX and primary cell characterization
PE anti-human Lambda	1-155-2	BD Biosciences, #642919	PDX and primary cell characterization
BB515 anti-human CD19	HIB19	BD Biosciences, #564456	PDX and primary cell characterization
APC anti-mouse CD45	30-F11	BioLegend, #103112	PDX and primary cell characterization
BV421 anti-human CD27	M-T271	BD Biosciences, #562513	B cell characterization
APC anti-human CD38	HIT2	BD Biosciences, #560980	B cell characterization
Pe-Cy7 anti-human IgD	IA6-2	BD Biosciences, #561314	B cell characterization
BV711 anti-human CD24	ML5	BD Biosciences, #563401	B cell characterization
PE anti-human CD138	MI15	BD Biosciences, #561704	B cell characterization
PerCP-Cy5.5 anti-human CD86	2331	BD Biosciences, #561129	B cell characterization
PE anti-human CD86	IT2.2	BD Biosciences, #555665	B cell characterization
AF700 anti-human HLA-DR	G46-6	BD Biosciences, # 560743	B cell characterization
Zombie Aqua Fixable Viability Kit		BioLegend, #423102	PDX, primary cell and B cells characterization

REFERENCES AND NOTES

1. J. Guck, S. Schinkinger, B. Lincoln, F. Wottawah, S. Ebert, M. Romeyke, D. Lenz, H. M. Erickson, R. Ananthakrishnan, D. Mitchell, J. Käs, S. Ulvick, C. Bilby, Optical deformability as an inherent cell marker for testing malignant transformation and metastatic competence. *Biophys. J.* **88**, 3689–3698 (2005).

2. M. Godin, F. F. Delgado, S. Son, W. H. Grover, A. K. Bryan, A. Tzur, P. Jorgensen, K. Payer, A. D. Grossman, M. W. Kirschner, S. R. Manalis, Using buoyant mass to measure the growth of single cells. *Nat. Methods* **7**, 387–390 (2010).

3. D. A. Fletcher, R. D. Mullins, Cell mechanics and the cytoskeleton. *Nature* **463**, 485–492 (2010).

4. S. Son, A. Tzur, Y. Weng, P. Jorgensen, J. Kim, M. W. Kirschner, S. R. Manalis, Direct observation of mammalian cell growth and size regulation. *Nat. Methods* **9**, 910–912 (2012).

5. O. Otto, P. Rosendahl, A. Mietke, S. Golfier, C. Herold, D. Klaue, S. Girardo, S. Pagliara, A. Ekpenyong, A. Jacobi, M. Wobus, N. Töpfner, U. F. Keyser, J. Mansfeld, E. Fischer-Friedrich, J. Guck, Real-time deformability cytometry: on-the-fly cell mechanical phenotyping. *Nat. Methods* **12**, 199–202 (2015).

6. K. R. Bashant, A. Vassallo, C. Herold, R. Berner, L. Menschner, J. Subburayalu, M. J. Kaplan, C. Summers, J. Guck, E. R. Chilvers, N. Toepfner, Real-time deformability cytometry reveals sequential contraction and expansion during neutrophil priming. *J. Leukoc. Biol.* **105**, 1143–1153 (2019).

7. T. W. Remmerbach, F. Wottawah, J. Dietrich, B. Lincoln, C. Wittekind, J. Guck, Oral cancer diagnosis by mechanical phenotyping. *Cancer Res.* **69**, 1728–1732 (2009).

8. H. T. K. Tse, D. R. Gossett, Y. S. Moon, M. Masaeli, M. Sohsman, Y. Ying, K. Mislick, R. P. Adams, J. Rao, D. Di Carlo, Quantitative diagnosis of malignant pleural effusions by single-cell mechanophenotyping. *Sci. Transl. Med.* **5**, 212ra163 (2013).

9. W. Xu, R. Mezencev, B. Kim, L. Wang, J. McDonald, T. Sulchek, Cell stiffness is a biomarker of the metastatic potential of ovarian cancer cells. *PLOS ONE* **7**, e46609 (2012).

10. R. J. Kimmerling, S. M. Prakadan, A. J. Gupta, N. L. Calistri, M. M. Stevens, S. Olcum, N. Cermak, R. S. Drake, K. Pelton, F. De Smet, K. L. Ligon, A. K. Shalek, S. R. Manalis, Linking single-cell measurements of mass, growth rate, and gene expression. *Genome Biol.* **19**, 207 (2018).

11. J. O. Armitage, D. L. Longo, Mantle-Cell Lymphoma. *N. Engl. J. Med.* **386**, 2495–2506 (2022).

12. C. López, E. Silkenstedt, M. Dreyling, S. Beà, Biological and clinical determinants shaping heterogeneity in mantle cell lymphoma. *Blood Adv.* **8**, 3652–3664 (2024).

13. P. Martin, A. Chadburn, P. Christos, K. Weil, R. R. Furman, J. Ruan, R. Elstrom, R. Niesvizky, S. Ely, M. Diliberto, A. Melnick, D. M. Knowles, S. Chen-Kiang, M. Coleman, J. P. Leonard, Outcome of deferred initial therapy in mantle-cell lymphoma. *J. Clin. Oncol.* **27**, 1209–1213 (2009).

14. P. Abrisqueta, D. W. Scott, G. W. Slack, C. Steidl, A. Mottok, R. D. Gascoyne, J. M. Connors, L. H. Sehn, K. J. Savage, A. S. Gerrie, D. Villa, Observation as the initial management strategy in patients with mantle cell lymphoma. *Ann. Oncol.* **28**, 2489–2495 (2017).

15. A. Kumar, Z. Ying, A. Alperovich, A. Dogan, P. Hamlin, C. Moskowitz, J. Pichardo, C. Portlock, F. Sha, A. D. Zelenetz, Z. Zhang, E. Drill, K. Woo, A. Younes, Clinical presentation determines selection of patients for initial observation in mantle cell lymphoma. *Haematologica* **104**, e163–e166 (2019).

16. P. Jain, M. L. Wang, Mantle cell lymphoma in 2022-A comprehensive update on molecular pathogenesis, risk stratification, clinical approach, and current and novel treatments. *Am. J. Hematol.* **97**, 638–656 (2022).

17. P. Jain, M. Wang, High-risk MCL: recognition and treatment. *Blood* **145**, 683–695 (2025).

18. E. Hoster, M. Dreyling, W. Klapper, C. Gisselbrecht, A. van Hoof, H. C. Kluin-Nelemans, M. Pfreundschuh, M. Reiser, B. Metzner, H. Einsele, N. Peter, W. Jung, B. Wörmann, W.-D. Ludwig, U. Dührsen, H. Eimermacher, H. Wandt, J. Hasford, W. Hiddemann, M. Unterhalt, German Low Grade Lymphoma Study Group (GLSG), European Mantle Cell Lymphoma Network, A new prognostic index (MIPI) for patients with advanced-stage mantle cell lymphoma. *Blood* **111**, 558–565 (2008).

19. P. Jain, M. Wang, Blastoid mantle cell lymphoma. *Hematol. Oncol. Clin. North Am.* **34**, 941–956 (2020).

20. G. Scheubeck, L. Jiang, O. Hermine, H. C. Kluin-Nelemans, C. Schmidt, M. Unterhalt, A. Rosenwald, W. Klapper, A. Evangelista, M. Ladetto, M. Jerkeman, S. Ferrero, M. Dreyling, E. Hoster, Clinical outcome of mantle cell lymphoma patients with high-risk disease (high-risk MIPI-c or high p53 expression). *Leukemia* **37**, 1887–1894 (2023).

21. E. Silkenstedt, M. Dreyling, Mantle cell lymphoma—Update on molecular biology, prognostication and treatment approaches. *Hematol. Oncol.* **41**, 36–42 (2023).

22. T. P. Burg, M. Godin, S. M. Knudsen, W. Shen, G. Carlson, J. S. Foster, K. Babcock, S. R. Manalis, Weighing of biomolecules, single cells and single nanoparticles in fluid. *Nature* **446**, 1066–1069 (2007).

23. J. Yu, Y. Zhang, S. M. Duquette, G. L. Yee, T. Dinter, T. R. Usherwood, W. Wu, T. Miettinen, S. Spranger, S. R. Manalis, Bimodal cell mass distribution separates CD8+ T cells into two distinct types with divergent differentiation dynamics. bioRxiv 615179 [Preprint] (2024). https://doi.org/10.1101/2024.09.26.615179.

24. J. H. Kang, T. P. Miettinen, L. Chen, S. Olcum, G. Katsikis, P. S. Doyle, S. R. Manalis, Noninvasive monitoring of single-cell mechanics by acoustic scattering. *Nat. Methods* **16**, 263–269 (2019).

25. E. C. Townsend, M. A. Murakami, A. Christodoulou, A. L. Christie, J. Köster, T. A. DeSouza, E. A. Morgan, S. P. Kallgren, H. Liu, S.-C. Wu, O. Plana, J. Montero, K. E. Stevenson, P. Rao,

R. Vadhi, M. Andreeff, P. Armand, K. K. Ballen, P. Barzaghi-Rinaudo, S. Cahill, R. A. Clark, V. G. Cooke, M. S. Davids, D. J. DeAngelo, D. M. Dorfman, H. Eaton, B. L. Ebert, J. Etchin, B. Firestone, D. C. Fisher, A. S. Freedman, I. A. Galinsky, H. Gao, J. S. Garcia, F. Garnache-Ottou, T. A. Graubert, A. Gutierrez, E. Halilovic, M. H. Harris, Z. T. Herbert, S. M. Horwitz, G. Inghirami, A. M. Intlekoffer, M. Ito, S. Izraeli, E. D. Jacobsen, C. A. Jacobson, S. Jeay, I. Jeremias, M. A. Kelliher, R. Koch, M. Konopleva, N. Kopp, S. M. Kornblau, A. L. Kung, T. S. Kupper, N. LaBoeuf, A. S. LaCasce, E. Lees, L. S. Li, A. T. Look, M. Murakami, M. Muschen, D. Neuberg, S. Y. Ng, O. O. Odejide, S. H. Orkin, R. R. Paquette, A. E. Place, J. E. Roderick, J. A. Ryan, S. E. Sallan, B. Shoji, L. B. Silverman, R. J. Soiffer, D. P. Steensma, K. Stegmaier, R. M. Stone, J. Tamburini, A. R. Thorner, P. van Hummelen, M. Wadleigh, M. Wiesmann, A. P. Weng, J. U. Wuerthner, D. A. Williams, B. M. Wollison, A. A. Lane, A. Letai, M. M. Bertagnolli, J. Ritz, M. Brown, H. Long, J. C. Aster, M. A. Shipp, J. D. Griffin, D. M. Weinstock, The public repository of xenografts enables discovery and randomized phase II-like trials in mice. *Cancer Cell* **29**, 574–586 (2016).

26. M. Dreyling, W. Klapper, S. Rule, Blastoid and pleomorphic mantle cell lymphoma: Still a diagnostic and therapeutic challenge! *Blood* **132**, 2722–2729 (2018).

27. S. H. Swerdlow, E. Campo, S. A. Pileri, N. L. Harris, H. Stein, R. Siebert, R. Advani, M. Ghielmini, G. A. Salles, A. D. Zelenetz, E. S. Jaffe, The 2016 revision of the World Health Organization classification of lymphoid neoplasms. *Blood* **127**, 2375–2390 (2016).

28. W. H. Grover, A. K. Bryan, M. Diez-Silva, S. Suresh, J. M. Higgins, S. R. Manalis, Measuring single-cell density. *Proc. Natl. Acad. Sci. U.S.A.* **108**, 10992–10996 (2011).

29. W. Wu, S. H. Ishamuddin, T. W. Quinn, S. Yerrum, Y. Zhang, L. L. Debaize, P.-L. Kao, S. M. Duquette, M. A. Murakami, M. Mohseni, K.-H. Chow, T. P. Miettinen, K. L. Ligon, S. R. Manalis, Measuring single-cell density with high throughput enables dynamic profiling of immune cell and drug response from patient samples. bioRxiv 591092 [Preprint] (2024). https://doi.org/10.1101/2024.04.25.591092.

30. S. Picelli, Å. K. Björklund, O. R. Faridani, S. Sagasser, G. Winberg, R. Sandberg, Smart-seq2 for sensitive full-length transcriptome profiling in single cells. *Nat. Methods* **10**, 1096–1098 (2013).

31. S. Picelli, O. R. Faridani, Å. K. Björklund, G. Winberg, S. Sagasser, R. Sandberg, Full-length RNA-seq from single cells using Smart-seq2. *Nat. Protoc.* **9**, 171–181 (2014).

32. F. Zhan, S. Colla, X. Wu, B. Chen, J. P. Stewart, W. M. Kuehl, B. Barlogie, J. D. Shaughnessy Jr, CKS1B, overexpressed in aggressive disease, regulates multiple myeloma growth and survival through SKP2- and p27Kip1-dependent and -independent mechanisms. *Blood* **109**, 4995–5001 (2007).

33. A. Roll-Mecak, The Tubulin Code in Microtubule Dynamics and Information Encoding. *Dev. Cell* **54**, 7–20 (2020).

34. S. Marchesi, F. Montani, G. Deflorian, R. D'Antuono, A. Cuomo, S. Bologna, C. Mazzoccoli, T. Bonaldi, P. P. Di Fiore, F. Nicassio, DEPDC1B Coordinates De-adhesion Events and Cell-Cycle Progression at Mitosis. *Dev. Cell* **31**, 420–433 (2014).

35. P. S. Winter, M. L. Ramseier, A. W. Navia, S. Saksena, H. Strouf, N. Senhaji, A. DenAdel, M. Mirza, H. H. An, L. Bilal, P. Dennis, C. S. Leahy, K. Shigemori, J. Galves-Reyes, Y. Zhang, F. Powers, N. Mulugeta, A. J. Gupta, N. Calistri, A. Van Scoyk, K. Jones, H. Liu, K. E. Stevenson, S. Ren, M. R. Luskin, C. P. Couturier, A. P. Amini, S. Raghavan, R. J. Kimmerling, M. M. Stevens, L. Crawford, D. M. Weinstock, S. R. Manalis, A. K. Shalek, M. A. Murakami, Mutation and cell state compatibility is required and targetable in Ph+ acute lymphoblastic leukemia minimal residual disease. bioRxiv 597767 [Preprint] (2024).

36. Y.-X. Liu, J. Wang, J. Guo, J. Wu, H. B. Lieberman, Y. Yin, DUSP1 is controlled by p53 during the cellular response to oxidative stress. *Mol. Cancer Res.* **6**, 624–633 (2008).

37. J. Zhang, D. Zhang, J. S. McQuade, M. Behbehani, J. Z. Tsien, M. Xu, c-fos regulates neuronal excitability and survival. *Nat. Genet.* **30**, 416–420 (2002).

38. V. Baldin, J. Lukas, M. J. Marcote, M. Pagano, G. Draetta, Cyclin D1 is a nuclear protein required for cell cycle progression in G1. *Genes Dev.* **7**, 812–821 (1993).

39. F. I. Montalto, F. De Amicis, Cyclin D1 in Cancer: A molecular connection for cell cycle control, adhesion and invasion in tumor and stroma. *Cells* **9**, 2648 (2020).

40. P. Scaffidi, T. Misteli, M. E. Bianchi, Release of chromatin protein HMGB1 by necrotic cells triggers inflammation. *Nature* **418**, 191–195 (2002).

41. M. Bustin, R. Reeves, "High-mobility-group chromosomal proteins: Architectural components that facilitate chromatin function," in *Progress in Nucleic Acid Research and Molecular Biology*, W. E. Cohn, K. Moldave, Eds. (Academic Press, 1996), vol. **54**, pp. 35–100b.

42. F. Yang, Y.-H. Wang, S.-Y. Dong, C.-Z. Chen, D.-P. Huang, MLF1IP promotes cells proliferation and apoptosis by regulating CyclinD1 in breast cancer. *Int. J. Clin. Exp. Pathol.* **10**, 11554–11562 (2017).

43. H. D. Yang, P.-J. Kim, J. W. Eun, Q. Shen, H. S. Kim, W. C. Shin, Y. M. Ahn, W. S. Park, J. Y. Lee, S. W. Nam, Oncogenic potential of histone-variant H2A.Z.1 and its regulatory role in cell cycle and epithelial-mesenchymal transition in liver cancer. *Oncotarget* **7**, 11412–11423 (2016).

44. M. Dong, J. Chen, Y. Deng, D. Zhang, L. Dong, D. Sun, H2AFZ Is a prognostic biomarker correlated to TP53 mutation and immune infiltration in hepatocellular carcinoma. *Front. Oncol.* **11**, 701736 (2021).

45. A. J. Marshall, A. K. Krahn, K. Ma, V. Duronio, S. Hou, TAPP1 and TAPP2 are targets of phosphatidylinositol 3-kinase signaling in B cells: sustained plasma membrane recruitment triggered by the B-cell antigen receptor. *Mol. Cell. Biol.* **22**, 5479–5491 (2002).

46. M. I. Merolle, M. Ahmed, K. Nomie, M. L. Wang, The B cell receptor signaling pathway in mantle cell lymphoma. *Oncotarget* **9**, 25332–25341 (2018).

47. P. Jares, D. Colomer, E. Campo, Molecular pathogenesis of mantle cell lymphoma. *J. Clin. Invest.* **122**, 3416–3423 (2012).

48. S. Yi, Y. Yan, M. Jin, S. Bhattacharya, Y. Wang, Y. Wu, L. Yang, E. Gine, G. Clot, L. Chen, Y. Yu, D. Zou, J. Wang, A. T. Phan, R. Cui, F. Li, Q. Sun, Q. Zhai, T. Wang, Z. Yu, L. Liu, W. Liu, R. Lyv, W. Sui, W. Huang, W. Xiong, H. Wang, C. Li, Z. Xiao, M. Hao, J. Wang, T. Cheng, S. Bea, A. F. Herrera, A. Danilov, E. Campo, V. N. Ngo, L. Qiu, L. Wang, Genomic and transcriptomic profiling reveals distinct molecular subsets associated with outcomes in mantle cell lymphoma. *J. Clin. Invest.* **132**, e153283 (2022).

49. V. C. Hecht, L. B. Sullivan, R. J. Kimmerling, D.-H. Kim, A. M. Hosios, M. A. Stockslager, M. M. Stevens, J. H. Kang, D. Wirtz, M. G. Vander Heiden, S. R. Manalis, Biophysical changes reduce energetic demand in growth factor-deprived lymphocytes. *J. Cell Biol.* **212**, 439–447 (2016).

50. M. Ming, W. Wu, B. Xie, M. Sukhanova, W. Wang, S. Kadri, S. Sharma, J. Lee, S. Shacham, Y. Landesman, N. Maltsev, P. Lu, Y. L. Wang, XPO1 inhibitor selinexor overcomes intrinsic ibrutinib resistance in mantle cell lymphoma via nuclear retention of IκB. *Mol. Cancer Ther.* **17**, 2564–2574 (2018).

51. W. Wu, W. Wang, C. A. Franzen, H. Guo, J. Lee, Y. Li, M. Sukhanova, D. Sheng, G. Venkataraman, M. Ming, P. Lu, A. Gao, C. Xia, J. Li, L. L. Zhang, V. C. Jiang, M. L. Wang, J. Andrade, X. Zhou, Y. L. Wang, Inhibition of B-cell receptor signaling disrupts cell adhesion in mantle cell lymphoma via RAC2. *Blood Adv.* **5**, 185–197 (2021).

52. J. A. Burger, Bruton tyrosine kinase inhibitors: Present and future. *Cancer J.* **25**, 386–393 (2019).

53. J. Lee, M. E. Robinson, N. Ma, D. Artadji, M. A. Ahmed, G. Xiao, T. Sadras, G. Deb, J. Winchester, K. N. Cosgun, H. Geng, L. N. Chan, K. Kume, T. P. Miettinen, Y. Zhang, M. A. Nix, L. Klemm, C. W. Chen, J. Chen, V. Khairnar, A. P. Wiita, A. Thomas-Tikhonenko, M. Farzan, J. U. Jung, D. M. Weinstock, S. R. Manalis, M. S. Diamond, N. Vaidehi, M. Müschen, IFITM3 functions as a PIP3 scaffold to amplify PI3K signalling in B cells. *Nature* **588**, 491–497 (2020).

54. M. Fichtner, M. Dreyling, M. Binder, M. Trepel, The role of B cell antigen receptors in mantle cell lymphoma. *J. Hematol. Oncol.* **10**, 164 (2017).

55. K. Saijo, C. Schmedt, I.-H. Su, H. Karasuyama, C. A. Lowell, M. Reth, T. Adachi, A. Patke, A. Santana, A. Tarakhovsky, Essential role of Src-family protein tyrosine kinases in NF-kappaB activation during B cell development. *Nat. Immunol.* **4**, 274–279 (2003).

56. A. I. Kim, P. Armand, R. A. Redd, M. Forsyth, P. Branch, S. Pazienza, L. Brennan, V. Patterson, S. M. Waisgerber, R. W. Merryman, D. C. Fisher, C. E. Ryan, I. E. Ahn, J. L. Crombie, O. O. Odejide, A. LaCasce, C. A. Jacobson, E. Jacobsen, E. M. Parry, M. S. Davids, J. R. Brown, A. S. Freedman, P. A. Riedell, M. A. Murakami, Phase I safety and preliminary efficacy of acalabrutinib, venetoclax, and obinutuzumab (AVO) in patients with relapsed/refractory mantle cell lymphoma. *Blood* **142**, 3031 (2023).

57. R. J. Kimmerling, M. M. Stevens, S. Olcum, A. Minnah, M. Vacha, R. LaBella, M. Ferri, S. C. Wasserman, J. Fujii, Z. Shaheen, S. Sundaresan, D. Ribadeneyra, D. S. Jayabalan, S. Agte, A. Aleman, J. A. Criscitiello, R. Niesvizky, M. R. Luskin, S. Parekh, C. A. Rosenbaum, A. Tamrazi, C. A. Reid, A pipeline for malignancy and therapy agnostic assessment of cancer drug response using cell mass measurements. *Commun. Biol.* **5**, 1295 (2022).

58. P. A. Thompson, C. S. Tam, Pirtobrutinib: a new hope for patients with BTK inhibitor–refractory lymphoproliferative disorders. *Blood* **141**, 3137–3142 (2023).

59. C. Tam, P. A. Thompson, BTK inhibitors in CLL: Second-generation drugs and beyond. *Blood Adv.* **8**, 2300–2309 (2024).

60. M. S. Davids, B. L. Lampson, S. Tyekucheva, Z. Wang, J. C. Lowney, S. Pazienza, J. Montegaard, V. Patterson, M. Weinstock, J. L. Crombie, S. Y. Ng, A. I. Kim, C. A. Jacobson, A. S. LaCasce, P. Armand, J. E. Arnason, D. C. Fisher, J. R. Brown, Acalabrutinib, venetoclax, and obinutuzumab as frontline treatment for chronic lymphocytic leukaemia: a single-arm, open-label, phase 2 study. *Lancet Oncol.* **22**, 1391–1402 (2021).

61. M. S. Davids, C. E. Ryan, B. L. Lampson, Y. Ren, S. Tyekucheva, S. M. Fernandes, J. L. Crombie, A. I. Kim, M. Weinstock, J. Montegaard, H. A. Walker, C. Greenman, V. Patterson, C. A. Jacobson, A. S. LaCasce, P. Armand, D. C. Fisher, S. Lo, A. J. Olszewski, J. E. Arnason, I. E. Ahn, J. R. Brown, Phase II study of acalabrutinib, venetoclax, and obinutuzumab in a treatment-naïve chronic lymphocytic leukemia population enriched for high-risk disease. *J. Clin. Oncol.* **43**, 788–799 (2025).

62. I. A. Hatton, E. D. Galbraith, N. S. C. Merleau, T. P. Miettinen, B. M. Smith, J. A. Shander, The human cell count and size distribution. *Proc. Natl. Acad. Sci. U.S.A.* **120**, e2303077120 (2023).

63. M. R. Luskin, M. A. Murakami, S. R. Manalis, D. M. Weinstock, Targeting minimal residual disease: a path to cure? *Nat. Rev. Cancer* **18**, 255–263 (2018).

64. T. P. Miettinen, J. H. Kang, L. F. Yang, S. R. Manalis, Mammalian cell growth dynamics in mitosis. *eLife* **8**, e44700 (2019).
